# Crystal structure of the 1:2 adduct of bis­(piperidinium) sulfate and 1,3-di­methyl­thio­urea

**DOI:** 10.1107/S2056989017004820

**Published:** 2017-04-04

**Authors:** Cindy Döring, Julian F. D. Lueck, Peter G. Jones

**Affiliations:** a Institut für Anorganische und Analytische Chemie, Technische Universität Braunschweig, Postfach 3329, D-38023 Braunschweig, Germany

**Keywords:** crystal structure, hydrogen bonds, piperidine, sulfate, di­methyl­thio­urea

## Abstract

In the title compound, 2C_5_H_12_N^+^·SO_4_^2−^·2C_3_H_8_N_2_S, the C=S groups of the two independent 1,3-di­methyl­urea mol­ecules and the sulfur atom of the anion lie on twofold axes. The packing is centred on bis­(piperidinium) sulfate ribbons parallel to the *c* axis; the cations are hydrogen bonded to the sulfate by N—H⋯O and C—H⋯O inter­actions. The 1,3-di­methyl­urea mol­ecules are also hydrogen bonded to sulfate O atoms, and project outwards from the ribbon parallel to the *b* axis.

## Abstract 2

The packing is centred on bis­(piperidinium) sulfate ribbons parallel to the *c* axis; the cations are hydrogen bonded to the sulfate by N—H⋯O and C—H⋯O inter­actions. The 1,3-di­methyl­urea mol­ecules are also hydrogen bonded to sulfate O atoms, and project outwards from the ribbon parallel to the *b* axis.

## Chemical context   

We are inter­ested in the structures of adducts of urea and thio­urea, and simple derivatives of these compounds, with neutral mol­ecules. We have published two reports on adducts of dioxane and morpholine with various methyl­thio­ureas (Jones *et al.*, 2013 ▸; Taouss & Jones, 2016 ▸). In the course of our current investigations, we attempted to obtain adducts of methyl­thio­ureas with piperidine, although mono­amines are not good adduct partners for ureas and thio­ureas. Indeed, no simple adducts were obtained. In one case, however, we overlayered a solution of 1,3-di­methyl­thio­urea (1,3-DMT) in piperidine with diethyl ether and obtained colourless crystals, the structure of which is reported here.
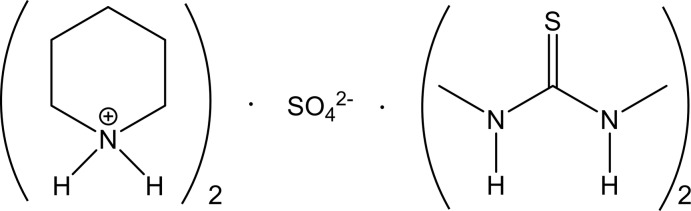


## Structural commentary   

**Figure 1:**
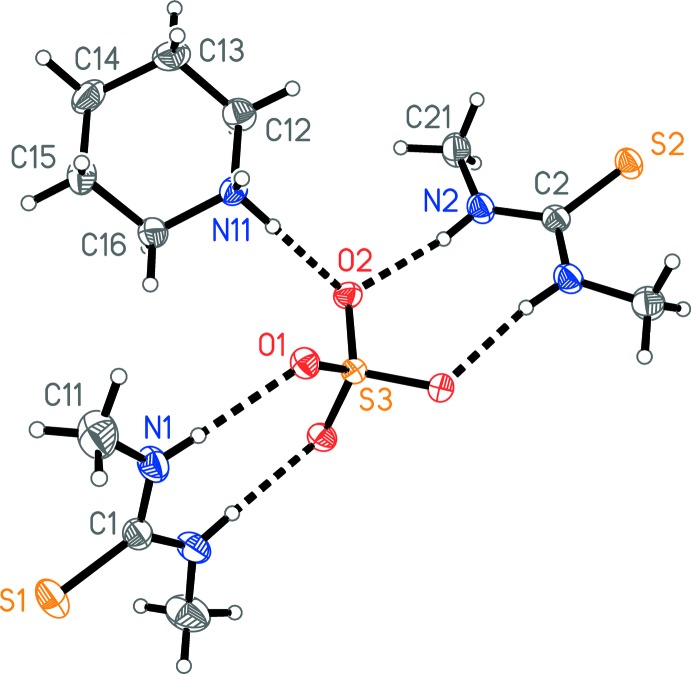
The structure of the title compound in the crystal. Only the asymmetric unit is labelled. Displacement ellipsoids represent 50% probability levels. The dashed lines represent hydrogen bonds.

The crystals proved to be a 1:2 adduct of bis­(piperidinium) sulfate and 1,3-DMT (Fig. 1 ▸), with the sulfate anion presumably generated by partial hydrolysis and/or decomposition of the 1,3-DMT under the influence of peroxides in the ether. The C=S bonds of both 1,3-DMT mol­ecules lie along twofold axes. The sulfate sulfur atom also lies on a twofold axis. The piperidine lies on a general position. Mol­ecular dimensions may be regarded as normal. Both 1,3-DMT mol­ecules are essentially planar (r.m.s. deviation of non-H atoms: 0.004 and 0.010 Å). Both NH functions of each 1,3-DMT are *trans* to the C=S double bond across the respective C—N bond (associated with the hydrogen-bonding pattern, see below), so that the methyl groups are *cis*, with C_meth­yl_—N—C=S torsion angles are close to zero [C11—N1—C1—S1 = 0.9 (2)° and S2—C2—N2—C21 = −2.05 (17)°]. Free 1,3-DMT crystallizes with four independent mol­ecules, each of which has one NH group *cis* and one *trans* to C=S, but the structure is severely disordered (Jones *et al.*, 2013 ▸).

## Supra­molecular features   

**Figure 2:**
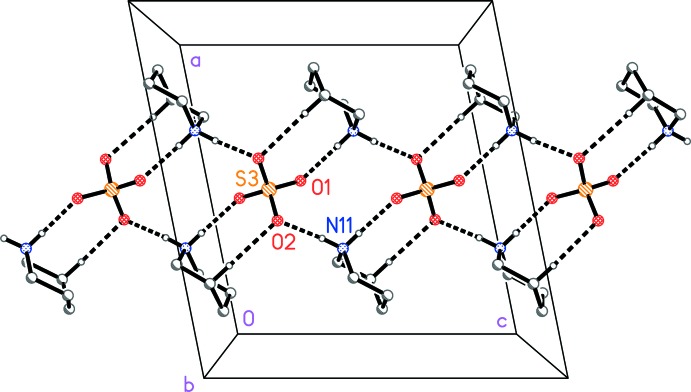
Packing diagram of the title compound: the bis­(piperidinium) sulfate substructure viewed parallel to the *b* axis. Dashed lines represent hydrogen bonds. Hydrogen atoms not involved in hydrogen bonds are omitted for clarity.

The packing is based on bis­(piperidinium) sulfate ribbons parallel to the *c* axis in the region *x*, *y* ≃ 1/2 (Fig. 2 ▸) and also at *x*, *y* ≃ 0, *etc*.; the cations are hydrogen bonded to the sulfate by N—H⋯O inter­actions, as expected, but also by a short inter­action C15—H15*A*⋯O2 (Table 1 ▸). Each pair of successive sulfate ions in the ribbon is bridged by two piperidinium cations. The 1,3-DMT mol­ecules are also hydrogen bonded to sulfate oxygens (Figs. 1 ▸ and 3 ▸); each 1,3-DMT bridges two oxygens of the same anion and projects outwards from the ribbons parallel to the *b* axis. In the presence of the sulfate oxygen atoms as strong hydrogen-bond acceptors, the 1,3-DMT sulfur atoms do not accept any classical hydrogen bonds.

## Database survey   

A search of the Cambridge Database (Version 1.19; Groom *et al.*, 2016 ▸) found three adducts of 1,3-DMT, excluding metal complexes. The 1:2 adduct between 18-crown-6 and 1,3-DMT (Weber, 1983 ▸) also displays a *trans* geometry for both NH functions, but the 1:2 adduct between 1,4-dioxane and 1,3-DMT (Jones *et al.*, 2013 ▸) and a 1,3-DMT adduct of a 1,3-DMT-gold(I) complex (Eikens *et al.*, 1994 ▸) both have one NH function *cis* and one *trans*. Only one other piperidinium sulfate derivative was found, namely tris­(piperidinium) hydrogensulfate sulfate (Lukianova *et al.*, 2015 ▸).

## Synthesis and crystallization   

208 mg (2 mmol) 1,3-DMT were dissolved in 2 mL piperidine. The solution was overlayered with diethyl ether. Colourless needles formed overnight.

## Refinement   

Crystal data, data collection and structure refinement details are summarized in Table 2 ▸. The asymmetric unit was chosen such that the occupied twofold axis is 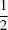, *y*, 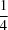. The NH hydrogen atoms were refined freely. The H atoms of the methyl groups were identified in a difference synthesis, idealized and refined as rigid groups allowed to rotate but not tip (C—H 0.98 Å, H—C—H 109.5°). Methyl­ene H atoms were included using a riding model starting from calculated positions (C—H 0.99 Å).

## Supplementary Material

Crystal structure: contains datablock(s) I, global. DOI: 10.1107/S2056989017004820/hg5484sup1.cif

Structure factors: contains datablock(s) I. DOI: 10.1107/S2056989017004820/hg5484Isup2.hkl

CCDC reference: 1540583

Additional supporting information:  crystallographic information; 3D view; checkCIF report

## Figures and Tables

**Figure 3:**
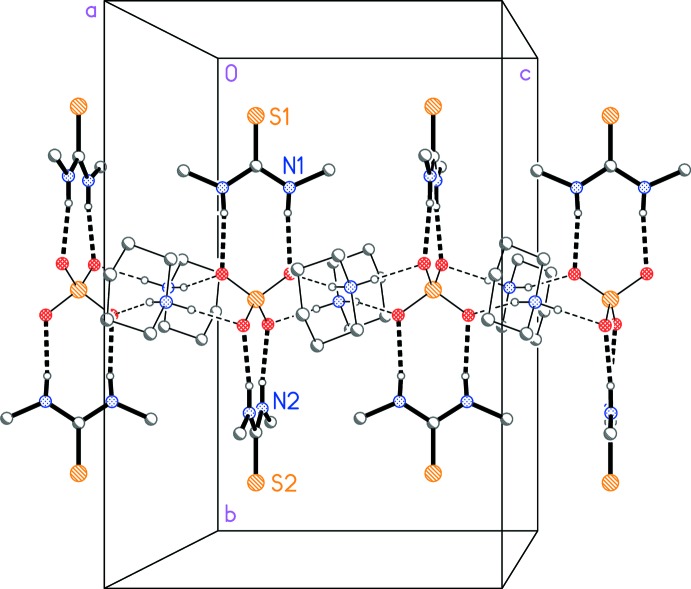
Packing diagram of the title compound: attachment of the thio­urea mol­ecules to the bis­(piperidinium) sulfate chain, viewed perpendicular to the *bc* plane. Dashed lines represent hydrogen bonds. Methyl­ene hydrogen atoms are omitted for clarity.

**Table 1:** Hydrogen-bond geometry (Å, °)

*D*—H⋯*A*	*D*—H	H⋯*A*	*D*⋯*A*	*D*—H⋯*A*
N1—H03⋯O1	0.86 (3)	2.06 (3)	2.9062 (18)	173 (2)
N2—H04⋯O2	0.89 (2)	2.00 (2)	2.8874 (17)	172 (2)
N11—H01⋯O1^i^	0.83 (2)	1.98 (2)	2.7953 (18)	167.4 (19)
N11—H02⋯O2	0.91 (2)	1.85 (2)	2.7589 (17)	176 (2)
C12—H12*A*⋯S1^ii^	0.99	2.94	3.8213 (17)	150
C15—H15*A*⋯O2^iii^	0.99	2.49	3.435 (2)	158

Symmetry codes: (i) 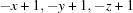; (ii) 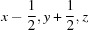; (iii) 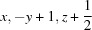.

**Table 2:** Experimental details

Crystal data	
Chemical formula	2C_5_H_12_N^+^·SO_4_^2−^·2C_3_H_8_N_2_S
*M*_r_	476.72
Crystal system, space group	Monoclinic, *C*2/*c*
Temperature (K)	100
*a*, *b*, *c* (Å)	12.5899 (5), 17.5691 (6), 11.8980 (5)
β (°)	101.326 (4)
*V* (Å^3^)	2580.52 (17)
*Z*	4
Radiation type	Cu *K*α
μ (mm^−1^)	2.89
Crystal size (mm)	0.25 × 0.05 × 0.02

Data collection	
Diffractometer	Oxford Diffraction Xcalibur, Atlas, Nova
Absorption correction	Multi-scan (*CrysAlis PRO*; Agilent, 2014 ▸)
*T*_min_, *T*_max_	0.514, 1.000
No. of measured, independent and observed [*I* > 2σ(*I*)] reflections	21112, 2701, 2295
*R*_int_	0.073
(sin θ/λ)_max_ (Å^−1^)	0.630

Refinement	
*R*[*F* ^2^ > 2σ(*F* ^2^)], *wR*(*F* ^2^), *S*	0.034, 0.084, 1.03
No. of reflections	2701
No. of parameters	152
H-atom treatment	H atoms treated by a mixture of independent and constrained refinement
Δρ_max_, Δρ_min_ (e Å^−3^)	0.21, −0.38

Computer programs: *CrysAlis PRO* (Agilent, 2014 ▸), *SHELXS97*, *SHELXL97* and *XP* (Sheldrick, 2008 ▸).

## supplementary crystallographic information

### Crystal data

**Table:**

2C_5_H_12_N^+^·SO_4_^2^^−^·2C_3_H_8_N_2_S	*F*(000) = 1032
*M**_r_* = 476.72	*D*_x_ = 1.227 Mg m^−^^3^
Monoclinic, *C*2/*c*	Cu *K*α radiation, λ = 1.54184 Å
Hall symbol: -C 2yc	Cell parameters from 6031 reflections
*a* = 12.5899 (5) Å	θ = 4.4–76.2°
*b* = 17.5691 (6) Å	µ = 2.89 mm^−^^1^
*c* = 11.8980 (5) Å	*T* = 100 K
β = 101.326 (4)°	Lath, colourless
*V* = 2580.52 (17) Å^3^	0.25 × 0.05 × 0.02 mm
*Z* = 4	

### Data collection

**Table:**

Oxford Diffraction Xcalibur, Atlas, Nova diffractometer	2701 independent reflections
Radiation source: sealed X-ray tube	2295 reflections with *I* > 2σ(*I*)
Mirror monochromator	*R*_int_ = 0.073
Detector resolution: 10.3543 pixels mm^-1^	θ_max_ = 76.2°, θ_min_ = 4.4°
ω scan	*h* = −15→15
Absorption correction: multi-scan (CrysAlisPro; Agilent, 2014)	*k* = −22→22
*T*_min_ = 0.514, *T*_max_ = 1.000	*l* = −14→14
21112 measured reflections	

### Refinement

**Table:**

Refinement on *F*^2^	Primary atom site location: structure-invariant direct methods
Least-squares matrix: full	Secondary atom site location: difference Fourier map
*R*[*F*^2^ > 2σ(*F*^2^)] = 0.034	Hydrogen site location: inferred from neighbouring sites
*wR*(*F*^2^) = 0.084	H atoms treated by a mixture of independent and constrained refinement
*S* = 1.03	*w* = 1/[σ^2^(*F*_o_^2^) + (0.0415*P*)^2^ + 0.6886*P*] where *P* = (*F*_o_^2^ + 2*F*_c_^2^)/3
2701 reflections	(Δ/σ)_max_ = 0.001
152 parameters	Δρ_max_ = 0.21 e Å^−^^3^
0 restraints	Δρ_min_ = −0.38 e Å^−^^3^

### Special details

**Table:**

Geometry. All esds (except the esd in the dihedral angle between two l.s. planes) are estimated using the full covariance matrix. The cell esds are taken into account individually in the estimation of esds in distances, angles and torsion angles; correlations between esds in cell parameters are only used when they are defined by crystal symmetry. An approximate (isotropic) treatment of cell esds is used for estimating esds involving l.s. planes.
Refinement. Refinement of F^2^ against ALL reflections. The weighted R-factor wR and goodness of fit S are based on F^2^, conventional R-factors R are based on F, with F set to zero for negative F^2^. The threshold expression of F^2^ > 2sigma(F^2^) is used only for calculating R-factors(gt) etc. and is not relevant to the choice of reflections for refinement. R-factors based on F^2^ are statistically about twice as large as those based on F, and R- factors based on ALL data will be even larger.

### Fractional atomic coordinates and isotropic or equivalent isotropic displacement parameters (Å^2^)

**Table:**

	*x*	*y*	*z*	*U*_iso_*/*U*_eq_	
S1	0.5000	0.15866 (3)	0.2500	0.03441 (16)	
N1	0.50629 (13)	0.29584 (8)	0.34615 (12)	0.0309 (3)	
H03	0.509 (2)	0.3444 (14)	0.342 (2)	0.043 (6)*	
C1	0.5000	0.25465 (12)	0.2500	0.0257 (4)	
C11	0.5151 (2)	0.26298 (12)	0.45902 (17)	0.0474 (5)	
H11A	0.4555	0.2271	0.4586	0.071*	
H11B	0.5116	0.3035	0.5148	0.071*	
H11C	0.5844	0.2361	0.4802	0.071*	
S2	0.5000	0.85910 (3)	0.2500	0.02659 (14)	
C2	0.5000	0.76309 (12)	0.2500	0.0225 (4)	
N11	0.32010 (11)	0.51380 (7)	0.44604 (12)	0.0221 (3)	
H01	0.3664 (18)	0.5141 (11)	0.5057 (19)	0.024 (5)*	
H02	0.3518 (18)	0.5281 (12)	0.387 (2)	0.033 (5)*	
C12	0.23562 (13)	0.57046 (9)	0.46151 (15)	0.0298 (3)	
H12A	0.1816	0.5756	0.3892	0.036*	
H12B	0.2697	0.6208	0.4809	0.036*	
C13	0.18007 (15)	0.54456 (11)	0.55658 (17)	0.0361 (4)	
H13A	0.1217	0.5809	0.5640	0.043*	
H13B	0.2330	0.5441	0.6301	0.043*	
C14	0.13203 (15)	0.46502 (11)	0.53210 (16)	0.0347 (4)	
H14A	0.0996	0.4481	0.5972	0.042*	
H14B	0.0739	0.4666	0.4627	0.042*	
C15	0.21891 (14)	0.40868 (10)	0.51400 (14)	0.0305 (4)	
H15A	0.2731	0.4031	0.5860	0.037*	
H15B	0.1855	0.3583	0.4936	0.037*	
C16	0.27441 (13)	0.43579 (9)	0.41936 (15)	0.0274 (3)	
H16A	0.3334	0.4001	0.4114	0.033*	
H16B	0.2217	0.4367	0.3457	0.033*	
S3	0.5000	0.50921 (3)	0.2500	0.01895 (12)	
O1	0.52691 (9)	0.46061 (6)	0.35314 (9)	0.0235 (2)	
O2	0.40655 (9)	0.55785 (6)	0.26031 (9)	0.0233 (2)	
N2	0.40879 (11)	0.72181 (8)	0.24440 (12)	0.0267 (3)	
H04	0.4138 (19)	0.6711 (13)	0.246 (2)	0.036 (6)*	
C21	0.30262 (14)	0.75398 (10)	0.24149 (17)	0.0331 (4)	
H21A	0.2771	0.7787	0.1673	0.050*	
H21B	0.3067	0.7916	0.3030	0.050*	
H21C	0.2521	0.7134	0.2522	0.050*	

### Atomic displacement parameters (Å^2^)

**Table:**

	*U*^11^	*U*^22^	*U*^33^	*U*^12^	*U*^13^	*U*^23^
S1	0.0329 (3)	0.0197 (3)	0.0475 (4)	0.000	0.0000 (3)	0.000
N1	0.0440 (8)	0.0225 (7)	0.0268 (7)	−0.0030 (6)	0.0080 (6)	0.0015 (5)
C1	0.0233 (10)	0.0230 (10)	0.0304 (12)	0.000	0.0047 (9)	0.000
C11	0.0703 (15)	0.0421 (11)	0.0295 (10)	−0.0070 (10)	0.0092 (10)	0.0057 (8)
S2	0.0272 (3)	0.0187 (2)	0.0334 (3)	0.000	0.0047 (2)	0.000
C2	0.0274 (11)	0.0228 (10)	0.0170 (9)	0.000	0.0038 (8)	0.000
N11	0.0200 (6)	0.0247 (6)	0.0219 (6)	−0.0015 (5)	0.0046 (6)	−0.0001 (5)
C12	0.0247 (8)	0.0268 (8)	0.0375 (9)	0.0050 (6)	0.0047 (7)	0.0004 (6)
C13	0.0282 (8)	0.0425 (10)	0.0408 (10)	−0.0003 (7)	0.0145 (8)	−0.0107 (8)
C14	0.0262 (8)	0.0466 (10)	0.0347 (9)	−0.0073 (7)	0.0141 (7)	−0.0030 (7)
C15	0.0303 (8)	0.0301 (8)	0.0304 (8)	−0.0075 (6)	0.0041 (7)	0.0025 (6)
C16	0.0250 (8)	0.0258 (8)	0.0323 (9)	−0.0036 (6)	0.0077 (6)	−0.0051 (6)
S3	0.0194 (2)	0.0182 (2)	0.0195 (2)	0.000	0.00437 (18)	0.000
O1	0.0262 (5)	0.0221 (5)	0.0217 (5)	−0.0004 (4)	0.0033 (4)	0.0014 (4)
O2	0.0224 (5)	0.0219 (5)	0.0270 (6)	0.0033 (4)	0.0085 (4)	0.0017 (4)
N2	0.0289 (7)	0.0200 (6)	0.0310 (7)	−0.0007 (5)	0.0056 (6)	0.0008 (5)
C21	0.0284 (8)	0.0282 (8)	0.0428 (10)	−0.0022 (6)	0.0071 (7)	0.0009 (7)

### Geometric parameters (Å, º)

**Table:**

S1—C1	1.687 (2)	C13—H13A	0.9900
N1—C1	1.3428 (19)	C13—H13B	0.9900
N1—C11	1.446 (2)	C14—C15	1.522 (3)
N1—H03	0.86 (3)	C14—H14A	0.9900
C1—N1^i^	1.3427 (19)	C14—H14B	0.9900
C11—H11A	0.9800	C15—C16	1.514 (2)
C11—H11B	0.9800	C15—H15A	0.9900
C11—H11C	0.9800	C15—H15B	0.9900
S2—C2	1.687 (2)	C16—H16A	0.9900
C2—N2	1.3487 (18)	C16—H16B	0.9900
C2—N2^i^	1.3488 (18)	S3—O2	1.4786 (10)
N11—C12	1.494 (2)	S3—O2^i^	1.4786 (10)
N11—C16	1.4961 (19)	S3—O1^i^	1.4787 (11)
N11—H01	0.83 (2)	S3—O1	1.4787 (11)
N11—H02	0.91 (2)	N2—C21	1.445 (2)
C12—C13	1.512 (3)	N2—H04	0.89 (2)
C12—H12A	0.9900	C21—H21A	0.9800
C12—H12B	0.9900	C21—H21B	0.9800
C13—C14	1.528 (3)	C21—H21C	0.9800
			
C1—N1—C11	123.85 (16)	C15—C14—C13	110.69 (14)
C1—N1—H03	119.0 (16)	C15—C14—H14A	109.5
C11—N1—H03	117.0 (16)	C13—C14—H14A	109.5
N1^i^—C1—N1	114.8 (2)	C15—C14—H14B	109.5
N1^i^—C1—S1	122.61 (10)	C13—C14—H14B	109.5
N1—C1—S1	122.61 (10)	H14A—C14—H14B	108.1
N1—C11—H11A	109.5	C16—C15—C14	110.50 (14)
N1—C11—H11B	109.5	C16—C15—H15A	109.5
H11A—C11—H11B	109.5	C14—C15—H15A	109.5
N1—C11—H11C	109.5	C16—C15—H15B	109.5
H11A—C11—H11C	109.5	C14—C15—H15B	109.5
H11B—C11—H11C	109.5	H15A—C15—H15B	108.1
N2—C2—N2^i^	114.94 (19)	N11—C16—C15	110.17 (13)
N2—C2—S2	122.53 (9)	N11—C16—H16A	109.6
N2^i^—C2—S2	122.53 (9)	C15—C16—H16A	109.6
C12—N11—C16	112.53 (13)	N11—C16—H16B	109.6
C12—N11—H01	106.8 (14)	C15—C16—H16B	109.6
C16—N11—H01	111.5 (13)	H16A—C16—H16B	108.1
C12—N11—H02	110.0 (14)	O2—S3—O2^i^	109.39 (9)
C16—N11—H02	107.4 (14)	O2—S3—O1^i^	110.26 (6)
H01—N11—H02	108.6 (19)	O2^i^—S3—O1^i^	108.74 (6)
N11—C12—C13	109.67 (14)	O2—S3—O1	108.74 (6)
N11—C12—H12A	109.7	O2^i^—S3—O1	110.26 (6)
C13—C12—H12A	109.7	O1^i^—S3—O1	109.46 (9)
N11—C12—H12B	109.7	C2—N2—C21	124.43 (14)
C13—C12—H12B	109.7	C2—N2—H04	118.6 (15)
H12A—C12—H12B	108.2	C21—N2—H04	116.9 (15)
C12—C13—C14	110.89 (15)	N2—C21—H21A	109.5
C12—C13—H13A	109.5	N2—C21—H21B	109.5
C14—C13—H13A	109.5	H21A—C21—H21B	109.5
C12—C13—H13B	109.5	N2—C21—H21C	109.5
C14—C13—H13B	109.5	H21A—C21—H21C	109.5
H13A—C13—H13B	108.0	H21B—C21—H21C	109.5
			
C11—N1—C1—N1^i^	−179.1 (2)	C13—C14—C15—C16	−55.77 (19)
C11—N1—C1—S1	0.9 (2)	C12—N11—C16—C15	−58.13 (18)
C16—N11—C12—C13	57.99 (18)	C14—C15—C16—N11	56.16 (18)
N11—C12—C13—C14	−56.3 (2)	N2^i^—C2—N2—C21	177.95 (17)
C12—C13—C14—C15	56.1 (2)	S2—C2—N2—C21	−2.05 (17)

Symmetry code: (i) −*x*+1, *y*, −*z*+1/2.

### Hydrogen-bond geometry (Å, º)

**Table:**

*D*—H···*A*	*D*—H	H···*A*	*D*···*A*	*D*—H···*A*
N1—H03···O1	0.86 (3)	2.06 (3)	2.9062 (18)	173 (2)
N2—H04···O2	0.89 (2)	2.00 (2)	2.8874 (17)	172 (2)
N11—H01···O1^ii^	0.83 (2)	1.98 (2)	2.7953 (18)	167.4 (19)
N11—H02···O2	0.91 (2)	1.85 (2)	2.7589 (17)	176 (2)
C12—H12*A*···S1^iii^	0.99	2.94	3.8213 (17)	150
C15—H15*A*···O2^iv^	0.99	2.49	3.435 (2)	158

Symmetry codes: (ii) −*x*+1, −*y*+1, −*z*+1; (iii) *x*−1/2, *y*+1/2, *z*; (iv) *x*, −*y*+1, *z*+1/2.
